# Experimental study of photon-counting CT neural network material decomposition under conditions of pulse pileup

**DOI:** 10.1117/1.JMI.8.1.013502

**Published:** 2021-01-09

**Authors:** Parker J. B. Jenkins, Taly Gilat Schmidt

**Affiliations:** Marquette University and Medical College of Wisconsin, Department of Biomedical Engineering, Milwaukee, United States

**Keywords:** neural networks, machine learning, photon counting, computed tomography, pulse pileup, spectral CT

## Abstract

**Purpose:** We investigated the performance of a neural network (NN) material decomposition method under varying pileup conditions.

**Approach:** Experiments were performed at tube current settings that provided count rates incident on the detector through air equal to 9%, 14%, 27%, 40%, and 54% of the maximum detector count rate. An NN was trained for each count-rate level using transmission measurements through known thicknesses of basis materials (PMMA and aluminum). The NN trained for each count-rate level was applied to x-ray transmission measurements through test materials and to CT data of a rod phantom. Material decomposition error was evaluated as the distance in basis material space between the estimated thicknesses and ground truth.

**Results:** There was no clear trend between count-rate level and material decomposition error for all test materials except neoprene. As an example result, Teflon error was 0.33 cm at the 9% count-rate level and 0.12 cm at the 54% count-rate level for the x-ray transmission experiments. Decomposition error increased with count-rate level for the neoprene test case, with 0.65-cm error at 9% count-rate level and 1.14-cm error at the 54% count-rate level. In the CT study, material decomposition error decreased with increasing incident count rate. For example, the material decomposition error for Teflon was 0.089, 0.066, 0.054 at count-rate levels of 14%, 27%, and 40%, respectively.

**Conclusions:** Results demonstrate over a range of incident count-rate levels that an NN trained at a specific count-rate level can learn the relationship between photon-counting spectral measurements and basis material thicknesses.

## 1 Introduction

Spectral CT with photon-counting detectors has demonstrated potential for improved image quality and quantitative material decomposition compared with conventional CT imaging methods.^1^ For example, photon-counting detectors have demonstrated improved spatial resolution,^2^ reduced electronic noise,^3^ and the ability to quantify K-edge contrast agents using three-material decomposition.^4^^–^^6^ However, photon-counting detectors are subject to nonideal effects, such as charge sharing and pulse pileup, which misrepresent the number and energy of detected photons.^7^^,^^8^ These spectral nonidealities can degrade the accuracy and precision of material decomposition estimates.

Material decomposition is an inverse problem that uses the acquired spectral measurements to estimate the composition of an unknown material in terms of basis components. Physics-based models and empirical models have been proposed to account for photon-counting detector nonidealities when performing material decomposition.^4^^,^^9^^–^^12^ A feedforward neural network (NN) material decomposition approach was previously investigated for photon-counting CT for both two-material decomposition^13^ and K-edge contrast agent imaging.^14^ Through calibration measurements, the NN learns the relationship between the measured counts data and the basis material thicknesses. In previous work, NN material decomposition was investigated under low tube current settings with negligible pileup effects.^13^^,^^14^ This previous work demonstrated that the NN can learn to compensate for the potential bias due to flux-independent spectral detector effects, such as charge sharing.

The purpose of this study was to investigate the performance of NN material decomposition under varying pulse-pileup conditions. Pulse-pileup occurs when more than one photon is absorbed by a detector element within a short time period such that the pulses generated by the photons overlap.^8^ Pulse pileup causes a loss of detected counts and detection of photons in the incorrect energy bins. The severity of pulse pileup effects increases as the count rate (counts per second per element) incident on the detector increases and with increasing detector element size. Pulse pileup is a complex effect that is difficult to incorporate into a physics-based forward model for material decomposition.

Previous studies investigated using an NN to correct the detected counts in each energy-bin measurement to compensate for nonideal effects. After correction, material decomposition can be performed using traditional methods assuming an ideal detector. Touch et al.^15^ demonstrated the ability of an NN to perform counts correction in experimental photon-counting data, but pileup effects were not explicitly studied. Feng et al.^16^ investigated an NN counts data correction across simulated pileup conditions.

The inversion from photon counts to basis material thicknesses is unstable, such that small errors or noise in the photon counts can lead to large errors in the basis material estimates. Therefore, errors introduced during the pileup-counts correction could be further amplified during the subsequent material decomposition. Instead of using an NN to correct the counts measurements prior to material decomposition, this study proposes using an NN to directly learn the relationship between the counts measurements and the basis material thicknesses in the presence of pileup effects. The NN thus accounts for pileup intrinsically as it performs material decomposition.

This paper investigates the question of whether an NN trained from and applied to data acquired at a specific incident count-rate level can perform accurate material decomposition, studied across a range of incident count-rate levels. This study also investigates whether the optimal NN architecture is sensitive to the incident count rate. A previous study demonstrated the ability of a material decomposition NN to achieve low bias and variance for data simulated with an idealized pileup model but without charge sharing or other spectral degradations.^17^ In this study, photon-counting experiments were performed to investigate the performance of NN material decomposition for both photon-counting x-ray transmission and CT measurements at varying incident count rates. Preliminary results of this study were presented in a conference proceedings paper.^18^

## 2 Methods and Materials

### 2.1 Neural Network Material Decomposition

The energy-dependent x-ray attenuation coefficient of a material can be represented as a linear combination of basis functions, which, for example, may represent the attenuation coefficients of two basis materials.^19^ The line integral through an object with energy-dependent x-ray attenuation distribution μ(x,y,z,E) measured along a ray path $\vec{r}$ by an x-ray transmission measurement can be represented as

$$\int \mu (x,y,z,E)d\overset{\to }{r}=\overset{M}{\underset{i=1}{\sum }}t_{i}f_{i}(E),$$

where M is the number of basis materials, $f_{i}(E)$ is the i’th energy-dependent basis attenuation function, and $t_{i}$ is the coefficient representing the contribution of the i’th basis function. If the basis functions are the linear attenuation functions of basis materials, the coefficients $t_{i}$ represent the equivalent thickness of each basis material. Material decomposition is the process of estimating the basis coefficients from the measured spectral data, which for photon-counting CT are the measured energy-bin counts. By performing this material decomposition for each measured ray path, basis material sinograms are created, from which basis material maps can be reconstructed using conventional CT reconstruction approaches.

**Fig. 1:**
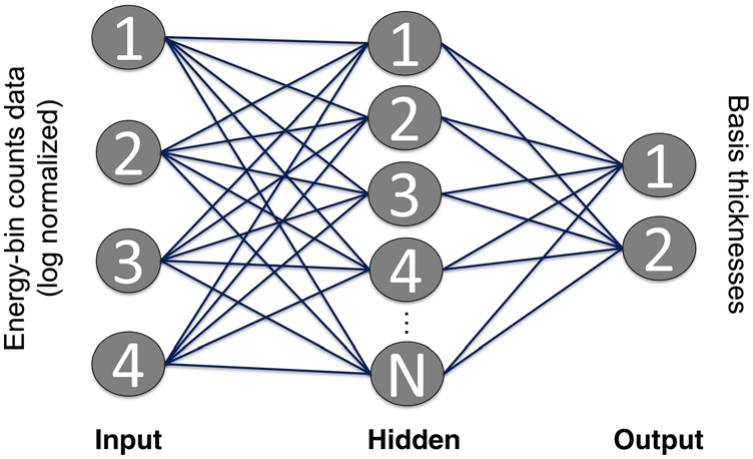
Overview of proposed NN material decomposition estimator. The NN consists of an input layer (log normalized energy bin counts data), an adjustable hidden layer, and an output layer (basis material thicknesses). The number of nodes in the hidden layer was determined using a leave-one-out cross validation study.

This study used an NN to estimate the equivalent thicknesses through two basis materials, $t_{1}$ and $t_{2}$ from the measured photon-counting spectral measurements. A feedforward NN with a single hidden layer, as shown in Fig. 1, was implemented as in previous work^13^^,^^14^ using the Python Scikit-Learn module.^20^ A network with one hidden layer was used for this regression problem, as theoretically a single-layer network is sufficient to approximate any function. The inputs to the NN are the log normalized energy-bin counts data $p_{k}$, which are calculated from the number of counts detected in energy bin k in the presence object, $n_{k}$, and in the absence of the object, $n_{k,0}$:

$$p_{k}=-\ln(\frac{n_{k}}{n_{k,0}}).$$

The energy bin data are input to the NN after log normalization so that the NN does not need to learn the approximate exponential relationship between the basis material thicknesses and the counts measurements. The number of elements in the hidden layer was investigated as described in Sec. 2.4. The NN outputs the estimated thicknesses of the two basis materials.

The NN used a sigmoid activation function, an L2 regularization parameter of 0.001, and the limited memory Broyden–Fletcher–Goldfarb–Shanno algorithm for network training.^21^ The sigmoid activation was selected because it is commonly used for shallow network architectures for which computation time is not a factor.^22^ The NN was trained with log-normalized transmission measurements through known thicknesses of the basis materials, as will be described in Sec. 2.3. During training, the NN learns the functional relationship between the basis material thicknesses and the energy-bin counts measurements. Pulse pileup effects are expected to change this functional relationship. This study trained an NN for a specific tube current setting corresponding to specific incident count rate through air and investigated the ability of the NN to learn the relationship between log-normalized energy-bin measurements and basis material thicknesses at that incident count-rate level. Once trained, the NN estimates the basis material pathlengths for an input set of log normalized energy-bin data measurements acquired at the same count-rate level used for training. Throughout this work, count-rate level refers to the counts per second per detector element incident on the detector through air.

### 2.2 Photon-Counting X-Ray Acquisition

Photon-counting spectral data were acquired on a bench-top spectral CT system with a CdTe photon-counting detector (DxRay, Northridge, California) and microfocus x-ray tube (L9181-02, Hamamatsu, Hamamatsu City, Japan). The detector consisted of an array of 4×64, 1.4  mm×1.0  mm elements with four comparator channels per element and a maximum count rate of 10^6^ counts per second per element as stated by the manufacturer. Data were acquired with a tube voltage of 90 kV and with four detected energy bins at thresholds of [20–45], [45–55], [55–65], [65–90] keV. To investigate the effects of pulse pileup, acquisitions were performed at five tube current settings of 0.02, 0.03, 0.06, 0.09, and 0.12 mA, with the detector placed 72 cm from the x-ray source. The acquisition time was adjusted for each tube current setting so that the tube current-time product was 0.456 mAs for all experiments, ensuring that the same number of photons were incident the detector for all experiments so that changes in noise were due to pileup conditions rather than differences in the number of photons reaching the detector.

**Fig. 2:**
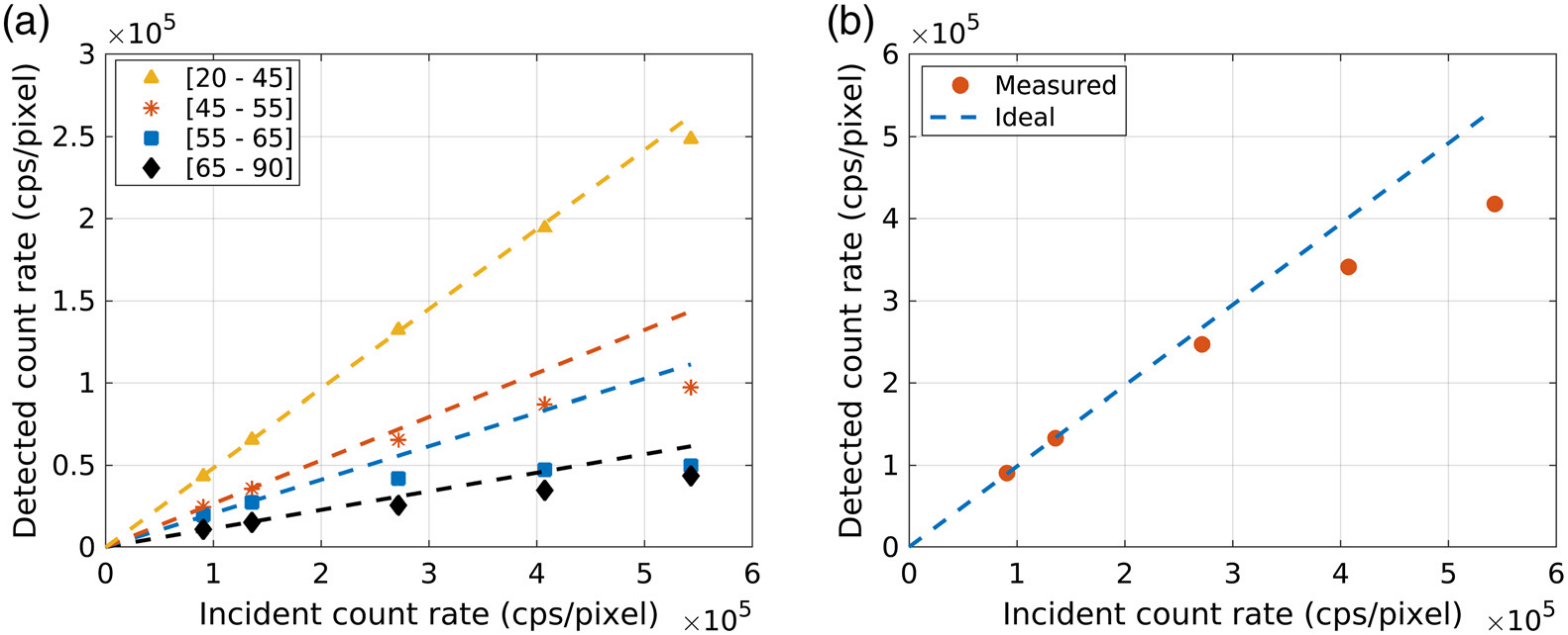
Detected count rate plotted against the count rate incident on the detector in air for (a) each energy bin and (b) the sum of all energy bins plotted for one example detector element. Each marker represents one of the five count-rate levels investigated in this study, corresponding to 9%, 14%, 27%, 40%, and 54% of the maximum detector count rate (10^6^). The ideal linear relationship between the detected and incident count rate, which would occur in the absence of pileup effects, is plotted for comparison. The ideal relationship was estimated by linear regression using the origin and the two lowest count rate data points. The plotted relationships are specific to the studied detector.

The tube current settings used in this study were approximately three orders of magnitude lower than what is used in clinical CT systems. The count rate capabilities of the bench-top detector were approximately three orders of magnitude lower than what is expected to be required for clinical photon-counting CT systems.^23^ To generalize the results of this study to the wide range of photon-counting detector capabilities, we report the studied cases as a percentage of the maximum detector count rate, so that the results are reported relative to the level of pileup instead of a detector-specific count-rate level. Figure 2 shows the detected count rate versus the incident count rate through air for the five investigated tube current settings, which corresponded to 9%, 14%, 27%, 40%, and 54% of the maximum detector count rate, demonstrating the level of pileup loss at each count-rate level and energy bin.

### 2.3 Neural Network Training

Calibration data for NN training were obtained by acquiring transmission measurements through a step wedge phantom consisting of 25 combinations of PMMA (0 to 4, 2.54 cm slabs) and aluminum (0 to 4, 0.64 cm slabs). Calibration data were acquired at each of the five count-rate levels shown in Fig. 2. The ground truth aluminum thicknesses were multiplied by four during network training to encourage similar levels of bias between the estimated PMMA and aluminum thicknesses. When the network was used for decomposition after training, the aluminum estimates were divided by four to compensate for this scaling. An NN was trained for each detector element and for each incident count-rate level. As proposed in our previous work, a transfer learning technique was used during training to reduce variations in bias across detector elements which can cause ring artifacts in reconstructed CT images.^14^ For each count-rate level, a network was first trained using the calibration energy-bin counts data from all detector elements. This produced an initial set of network weights that fit the average detector response. These weights were then used to initialize a network for each detector element. The element-specific networks were further trained using the calibration data from that specific detector element.

### 2.4 Selection of Number of Hidden Layer Elements

A leave-one-out cross-validation study was performed to select the number of nodes in the hidden layer and to investigate whether pulse pileup effects alter the optimal network configuration. The number of hidden-layer nodes was varied between 5 and 100. During each cross-validation run, the network was trained on 24 calibration measurements, with one calibration measurement left out for testing. The trained network was then used to predict the basis material thicknesses of the test calibration measurement. The calibration points at the extreme corners of the calibration space (0-cm PMMA, 0-cm aluminum) and (10.16-cm PMMA, 2.54-cm aluminum) were not used as testing datasets to prevent the need for the network to extrapolate beyond the calibrated space. The root mean squared error (RMSE) between the true basis material thicknesses and the thicknesses estimated by the NN was calculated across all cross-validation test data. The number of hidden elements for subsequent studies was selected based on the resulting RMSE, after which an NN was trained for each count-rate level using all 25 calibration measurements.

### 2.5 NN Evaluation Using Test Material X-Ray Transmission Measurements

To quantify the performance of the NN material decomposition across different pileup conditions, photon-counting x-ray transmission measurements were acquired through each of 3.81-cm-thick Teflon (8545K26, McMaster-Carr, Elmhurst, Illinois), 5.08-cm thick Delrin (8545K26, McMaster-Carr, Elmhurst, Illinois), and 2.54-cm-thick neoprene (9013K12, McMaster-Carr, Elmhurst, Illinois) using the acquisition protocol and count-rate levels described in Sec. 2.2. The transmission measurements were repeated five times for each test material and count-rate level. The log normalized energy-bin counts data at each count-rate level were input to the corresponding network trained for each detector element and count-rate level, resulting in the estimated basis material thicknesses for each count-rate level, detector element, and for each of the five trials.

The two basis material thicknesses returned by the NN estimator represent a point in two-dimensional (2D) material decomposition space. The 2D Euclidean distance, D, between the true basis material thickness coordinates, $t_{\mathrm{PMMA}}$ and $t_{\mathrm{Al}}$, and the basis material thicknesses estimated by the NN, t^PMMA, and t^Al, was calculated as a metric of material decomposition accuracy, as expressed in the equation below, which is also proportional to the RMSE. The ground truth material decomposition coefficients were estimated by simulating the polyenergetic photon-counting transmission measurements using the attenuation coefficient functions provided by the NIST XCOM database^23^ followed by maximum likelihood decomposition. The uncertainty of the NIST attenuation functions is estimated at 1% to 2%.^24^ This distance metric gives more weight to error in the basis material that has a greater contribution to the overall attenuation.

D=(tPMMA−t^PMMA)2+(tAl−t^Al)2.

The median, lower quartile, and upper quartile of the distance metric were calculated across the five trials and all detector elements for each count-rate level and for each of the Teflon, Delrin, and neoprene test materials. The ground truth decomposition values contain uncertainty due to potential impurities in the materials, uncertainty of NIST attenuation coefficients, and uncertainty introduced during material decomposition. As an example for the 3.81-cm Teflon test slab, introducing −2% bias in the density and −2% bias in the NIST attenuation coefficients causes 0.2-cm distance error in the material decomposition estimates. To further evaluate NN performance without these uncertainties, the distance error metric was calculated for three combinations of known thicknesses of PMMA and aluminum using results of the leave-one-out study. The tested PMMA and aluminum combinations were selected as those with composition most similar to each of the three test materials, as described in Table 1.

**Table 1:** Ground truth basis material thicknesses for the three test materials and for the three combinations of known thicknesses of PMMA and aluminum used to evaluate NN performance. The combinations of PMMA and aluminum were selected to be similar to the compositions of the test materials.

	Delrin	Combination 1	Teflon	Combination 2	Neoprene	Combination 3
PMMA (cm)	5.79	5.08	5.44	5.08	0.12	0.00
Al (cm)	0.08	0.00	0.49	0.635	0.56	0.635

### 2.6 NN Evaluation Using Photon-Counting CT Acquisitions

To more fully evaluate the NN performance across incident count-rate level, photon-counting CT data were acquired of a 6.35-cm-diameter PMMA phantom with 1.9-mm-diameter cylindrical inserts of LDPE (8754K46, McMaster-Carr, Elmhurst, Illinois), PMMA (8573K13, McMaster-Carr, Elmhurst, Illinois), and Teflon (8546K15, McMaster-Carr, Elmhurst, Illinois). CT data were acquired at 120 views at 14%, 27%, and 40% of the maximum detector count rate, with the scan time scaled at each tube current setting to maintain a constant tube-current-time-product across count-rate levels. At each view, the detector was translated to two positions to encompass the phantom field of view. The trained NN for each count-rate was applied to decompose the CT data into PMMA and aluminum basis sinograms, which were then reconstructed into basis images using filtered backprojection.

NN performance was evaluated by extracting ROIs within the Teflon, LDPE, and PMMA inserts in each of the resulting PMMA and aluminum basis images at each count-rate level. The median, lower quartile, and upper quartile of the distance metric were calculated within each ROI and count-rate level. The standard deviation of basis map values within the ROIs was also calculated.

## 3 Results

### 3.1 Selection of Number of Hidden Layer Elements

**Fig. 3:**
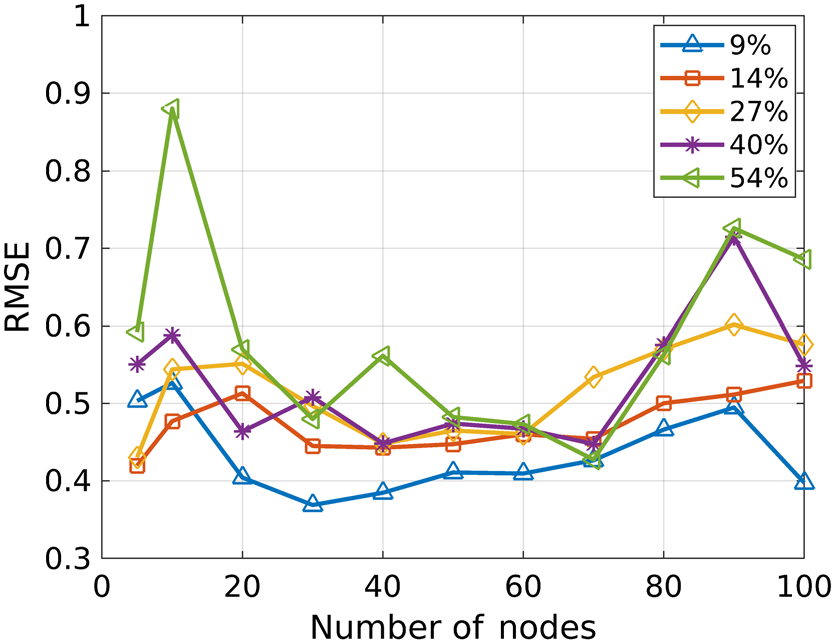
The results of the leave-one-out cross validation study are plotted for each of the studied detected count-rate levels. The detected count-rate levels are represented as percent of the maximum detector count rate. The RMSE of the validation test points, averaged across the validation trials and across all detector elements, are plotted for the different number of nodes in the hidden layer.

Figure 3 shows the RMSE resulting from the leave-one-out cross-validation study of the number of hidden-layer nodes. The RMSE, calculated by averaging all validation trials and detector elements, is plotted for each tested number of nodes and for each studied count-rate level. The results demonstrate that a hidden layer with 50 to 60 nodes generally provided low validation error for all count-rate levels, while 30 nodes provided lowest RMSE for the lowest count-rate level and 70 nodes provided lowest RMSE for the two higher count-rate levels. The lowest count-rate level (9% of maximum count rate) resulted in the lowest RMSE for all network architectures. The RMSE was similar across the other count-rate levels when 50 or 60 nodes were used. Based on these results, the number of hidden layer nodes in the NN was 30, 30, 40, 70, and 70 for the respective count-rate levels of 9%, 14%, 27%, 40%, and 54% in the subsequent study. A study was also performed using 50 hidden-layer nodes for each count-rate level, with results presented in the Appendix.

### 3.2 NN Evaluation Using Test Material X-Ray Transmission Measurements

**Fig. 4:**
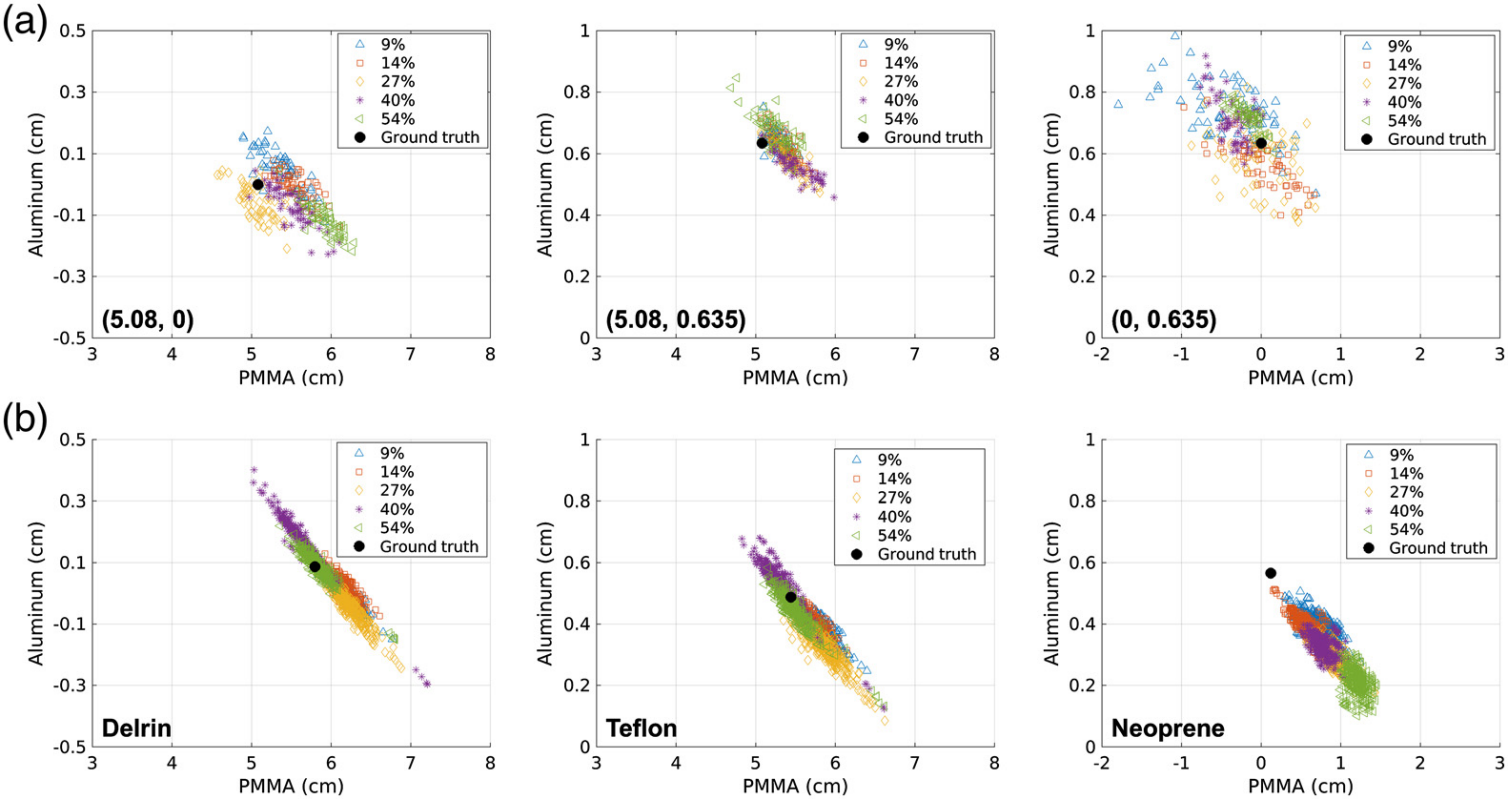
Material decomposition values estimated by the NN displayed in the 2-D material decomposition space. (a) The results of decomposing the known combinations of (PMMA, aluminum) thicknesses in the leave-one-out study. (b) Results from the test materials. Results are plotted for each test material and detector element, and, for the test materials, each of the five trials. Each marker type represents the detected count-rate level specified as the percent of the maximum detector count rate. The ground truth material decomposition coefficients are also plotted for each case.

Figure 4 shows the NN material decomposition estimates in the 2-D material decomposition space for each detector element, count-rate level, and trial. Results are presented for the test combinations of calibration materials (top row) and the test material slabs (bottom row). The ground truth material decomposition values are also plotted.

**Fig. 5:**
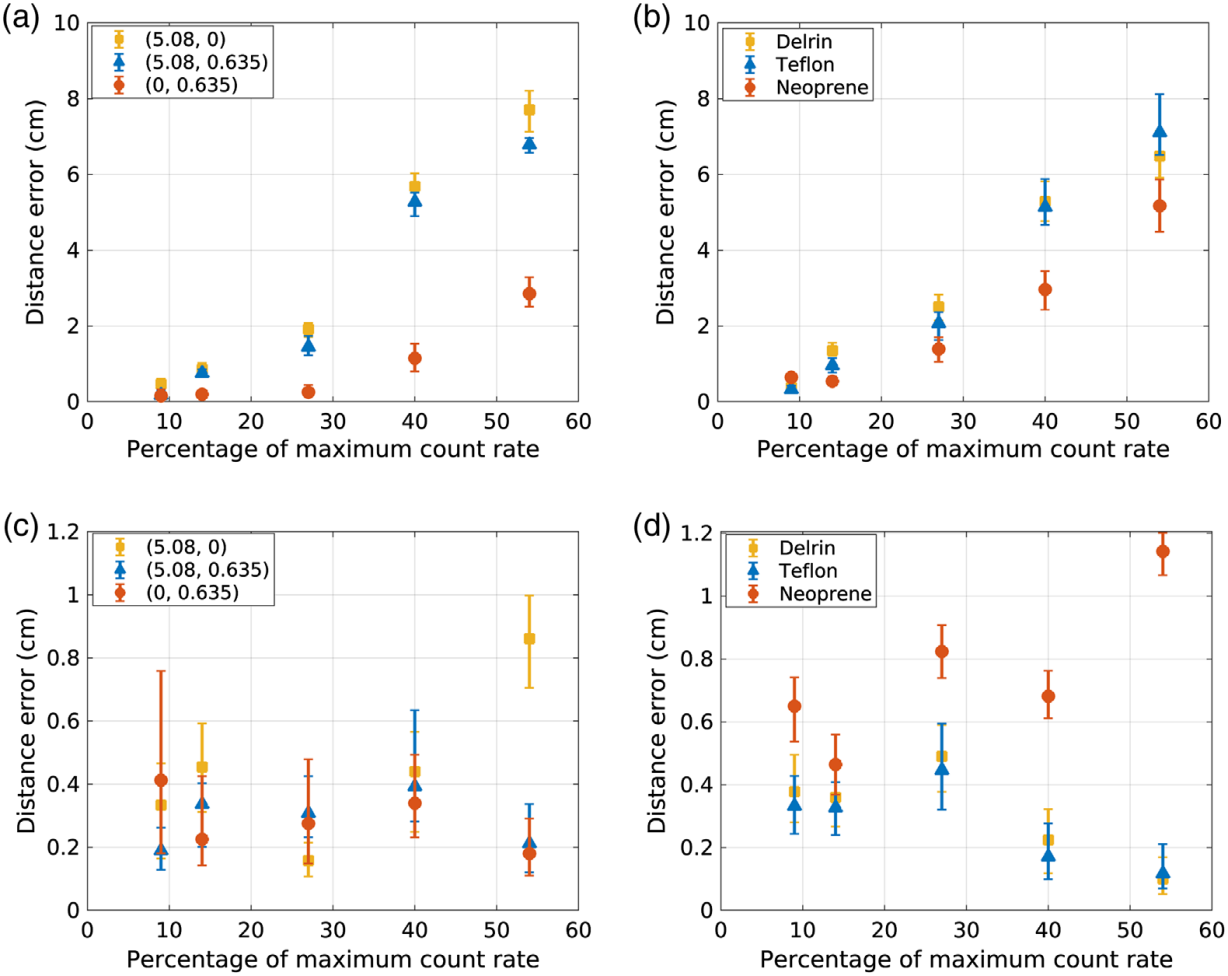
The top row displays the distance error plotted against detected count-rate level for an NN trained with data from the lowest detected count-rate level and then applied to data acquired at varying count rates for (a) (PMMA, Al) test combinations and (b) test materials. The bottom row displays the distance error resulting from NNs trained specifically for each count-rate level for (c) (PMMA, Al) test combinations and (b) test materials. The plotted points represent the median distance error across all detector elements and trials, while the error bars represent the upper and lower quartiles.

Figure 5 first plots the material decomposition distance error against detected count-rate level for an NN that was trained with data from only the lowest count-rate level (9%) and then applied to decompose data from all count-rate levels. While this approach is not recommended, these results demonstrate the magnitude of potential error due to pileup, which increases with increasing count-rate level. Figure 5 also plots the distance error that results from NNs trained specifically for each count-rate level, demonstrating the ability of the trained NNs to reduce error due to pileup. For example, the error in the Teflon material decomposition was 0.12 cm for the 54% count-rate level when using an NN trained specifically with data acquired at that count-rate level, compared with an error of 7.1 cm when the NN was trained with data from the 9% count-rate level.

As seen in Fig. 4, the incident count rate affected the relative contributions of basis material thicknesses estimated by the NNs. For example, PMMA had a larger contribution to the Teflon decomposition at low count rates than high count rates, while the aluminum contribution demonstrated the opposite trend. However, the overall distance error for the Teflon case remained fairly constant with incident count-rate level, with error of 0.33 cm at the 9% count-rate level and 0.12 at the 54% count-rate level. All NNs overestimated the PMMA contribution of neoprene while underestimating the aluminum contribution. The neoprene results demonstrated the highest overall error, with the error increasing with count-rate level. The neoprene error at the 9% count-rate level was 0.65 cm, compared with 1.14 cm at the 54% level for the NNs specifically trained at each count-rate level. While the error increased with count rate for the neoprene decomposition, the count-rate-specific NN considerably reduced the potential error due to pileup when compared with the 5.2-cm error for the 54% count-rate level when using an NN trained at 9% count-rate level. The test combination of 0.0-cm PMMA and 0.635-cm aluminum, which was close to neoprene in the decomposition space, did not show the trend of increasing error with increasing count rate. There was no clear trend between count-rate level and material decomposition error for Delrin, Teflon, or for the tested combinations of PMMA and aluminum. For materials other than neoprene, the differences in basis material decomposition were within the estimated 0.2-cm uncertainty of the ground truth values.

**Fig. 6:**
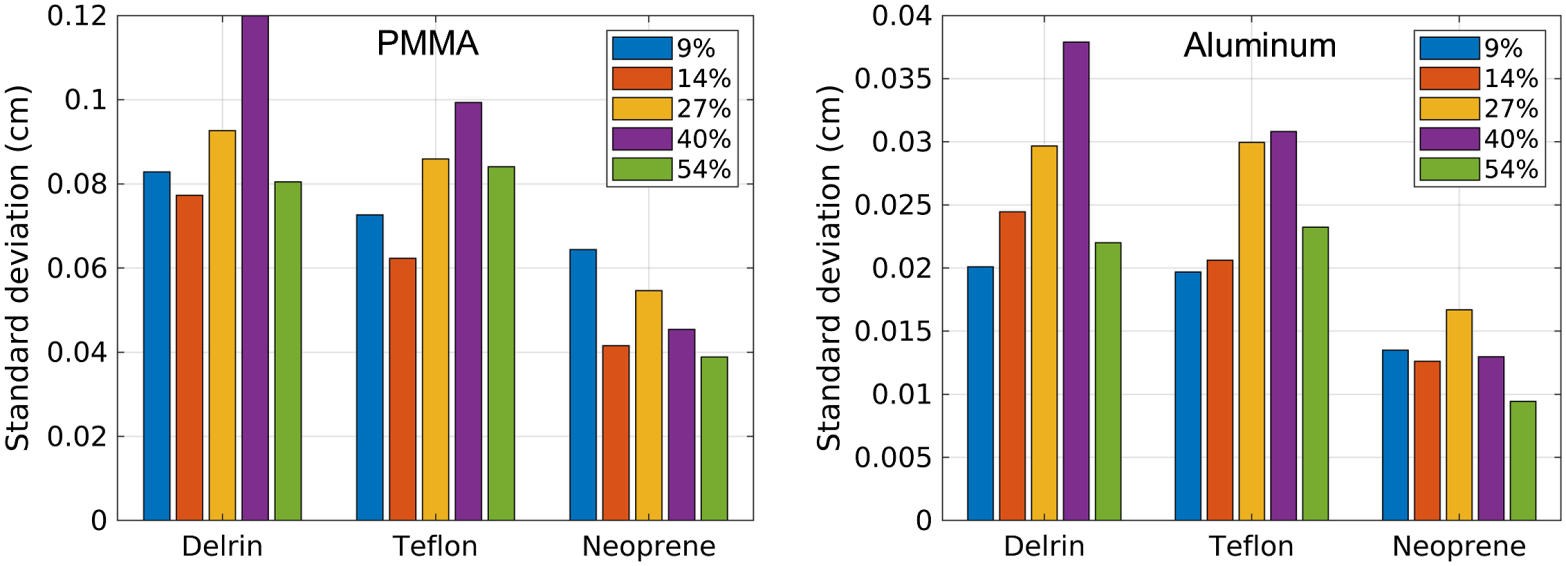
Standard deviation of the PMMA and aluminum basis material thicknesses estimated by a NN trained for each count-rate level. The standard deviation was calculated across the five trials and then averaged across detector elements for each test material.

Figure 6 shows the standard deviation of the basis material thicknesses estimated by the NN for each test material and count-rate level. To reduce the effects of detector element-to-element variations on the quantification of standard deviation, the standard deviation was calculated across the five trials and then averaged across detector elements. As seen in Fig. 6, the standard deviation of the NN estimates did not show a clear trend with count-rate level. The 40% count-rate level demonstrated the highest standard deviation, which may suggest that using 70 nodes in the hidden layer may not be optimal and may cause overfitting. The appendix evaluates the performance of NNs with 50 nodes in the hidden layer for each count-rate level. The results in the appendix demonstrate that using 50 nodes may be beneficial for the 40% and 54% count-rate levels.

### 3.3 NN Evaluation Using Photon-Counting CT Acquisitions

**Fig. 7:**
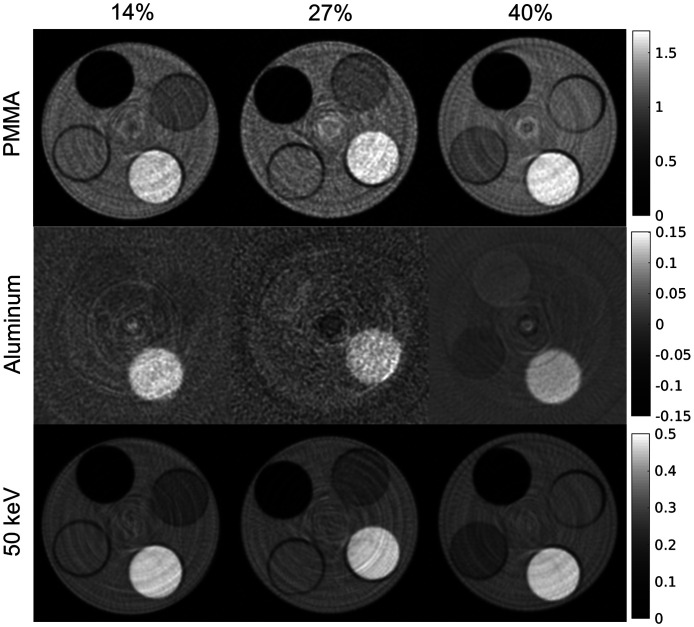
PMMA and aluminum basis images reconstructed from the basis sinograms that were estimated by the trained NN for each studied flux level. The rods, from brightest to darkest, are Teflon, PMMA, LDPE, and air. The 50-keV virtual monoenergetic image formed by the basis images is also shown for each flux level. The flux level is reported as the percentage of the maximum detector count rate.

**Fig. 8:**
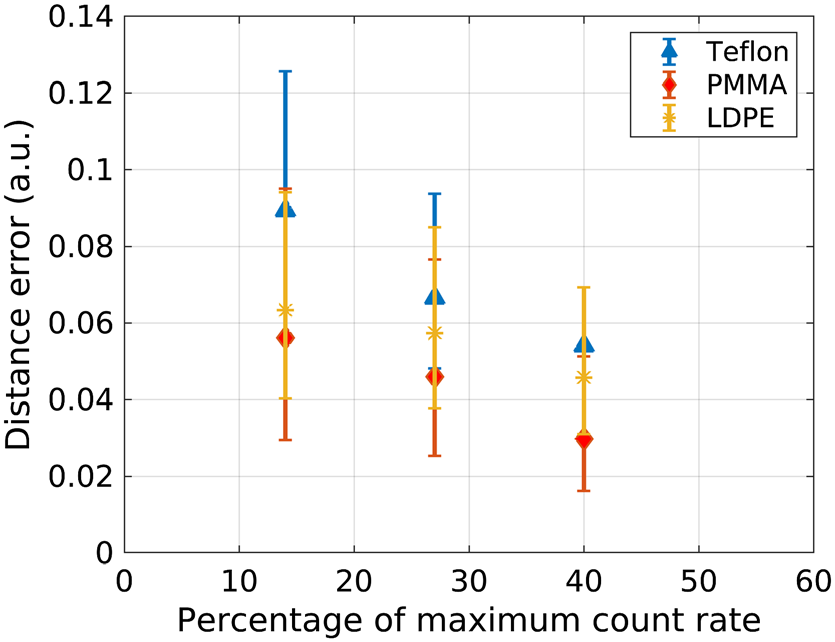
Euclidean distance error metric estimated in the Teflon, PMMA, and LDPE regions of the basis images shown in Fig. 7 for the three studied count-rate levels. The data points represent the median error in the ROI. The error bars represent the upper and lower quartile of the error in the ROI. The basis coefficients in the CT basis images represent the contribution of each basis material and are thus unitless.

The PMMA and aluminum basis images reconstructed from the basis sinograms estimated by the NNs are shown in Fig. 7 for incident count rates of 14%, 27%, and 40% of the maximum detector count rate. The number of hidden nodes used in the NN for the 14% and 27% count-rate levels was 30 and 40, respectively, as in the results presented in Sec. 3.2. As described in the Appendix, an NN architecture with 50 hidden-layer nodes was determined to be advantageous for the 40% count-rate level and was used for this study. The images in Fig. 7 demonstrate similar gray-level values despite the different pileup effects. Figure 8 shows the median distance error for ROIs within the Teflon, PMMA, and LDPE rods of the phantom. The quantitative error for all three rods decreased with increasing count-rate. For example, in the Teflon rod, the material decomposition error was 0.089, 0.066, 0.054 at count-rate levels of 14%, 27%, and 40%, respectively. The noise level in the basis images varied with count-rate level but did not show the trend of increased noise with count-rate level that would be expected due to dead time losses. For example, the standard deviation, which takes into account both noise and ring artifacts, was 0.012, 0.017, and 0.006 in the Teflon ROI of the aluminum basis image for the 14%, 27%, and 40% count-rate levels, respectively, and 0.054, 0.064, 0.043 in the Teflon ROI of the PMMA basis image.

## 4 Discussion

Pulse-pileup is known to degrade the spectral performance of photon-counting detectors by registering photons in incorrect energy bins and reducing the number of detected counts. Pulse pileup, therefore, changes the relationship between the basis material estimates and the measured photon-counting spectral measurements. The results of this study demonstrate that an NN can learn the relationship between the photon-counting energy-bin x-ray transmission measurements and the basis materials thicknesses even at high count-rate levels, thereby compensating for pulse pileup effects.

The cross-validation study evaluating the NN architecture demonstrated that an architecture with 30 nodes was optimal for the hidden layer at lower count-rate levels, while using 50 to 70 nodes provided lowest RMSE at higher count-rate levels. This result suggests that pulse pileup may increase the complexity of the function being fit by the network.

The plots in Fig. 4 show the results in the 2D material decomposition space. The Cartesian coordinates of the material decomposition space represent the contribution of the two basis materials. When considering the polar coordinates of the NN estimates, the angular coordinate represents the composition of the material, while the radial distance to the origin represents material thickness.^12^ As seen in Fig. 4, the basis coefficients output by the NN at different count-rate levels generally provide similar estimates of material thickness, while differing in their estimates of material composition. The neoprene test material demonstrated bias at all count-rate levels, with increasing error at higher count-rate levels. Neoprene decomposes into a higher component of aluminum than PMMA. The calibration test space included measurements of only aluminum but not measurements with more aluminum than PMMA. Therefore, the neoprene test case is an example of extrapolation of the network from the calibration space, which resulted in more error at higher count-rate levels. The decomposition of such materials may be improved in the future by increasing the calibration space.

Quantification of material decomposition error is confounded by uncertainties in the composition of the test materials, uncertainties in the NIST attenuation coefficient functions, and uncertainties in estimating the ground truth basis material coefficients. These uncertainties may account for some of the deviation from ground truth seen in the Fig. 4 scatter plots. With the exception of the neoprene test, the material decomposition error remained fairly consistent across count-rate level and within the uncertainty of the ground truth estimates when using NNs trained specifically for each count-rate level. The standard deviation of the material decomposition estimates did not show trends with count-rate level, despite the loss of counts due to pileup. The variance of NN estimates depends on data noise and model uncertainty.^25^ The reduced noise and error of material decomposition estimates at higher count-rate levels for some test materials may suggest that pileup does not increase model uncertainty at higher count-rate levels. This study used the same level of regularization for NN’s at all count-rate levels. It may be beneficial in future studies to investigate different levels of regularization for different count-rate levels.

Pulse pileup effects depend on detector characteristics, including detector deadtime, pulse shape, and element size. Smaller detector elements have less pileup effects but more charge sharing, both of which affect material decomposition. The results of this study demonstrate potential for NN decomposition in the presence of pileup effect but are specific to the studied detector because of these complex factors.

Twenty-five calibration measurements were used to train the NN in this study. Additional calibration measurements may help improve accuracy at all count-rate levels. However, the need for more calibration measurements must be balanced by the practicality of calibration procedures. This study proposes training an NN for each count-rate level, which may be a challenge for clinical CT when considering the range of tube currents used due to patient size variations and the use of continuous tube current modulation. To implement this method in conjunction with tube current modulation, the NN applied to each detector element at each projection view would depend on the tube current used for that view. At each tube current setting, a network would be trained across a range of basis material thicknesses, thus accounting for different patient sizes. Motivated by the results of this study, further research is underway to develop a single NN estimator to perform material decomposition across a range of count-rate levels. Efficient methods and phantoms are needed to train NNs across a range of incident count-rate levels.

## 5 Conclusions

NNs trained specifically for each count-rate level reduced the material decomposition error due to pileup and provided generally consistent error across a range of count-rate levels. The results of this study demonstrate that an NN can learn the relationship between the photon-counting energy-bin x-ray transmission measurements and the basis materials thicknesses even at high count-rate levels, thereby compensating for pulse pileup effects.

## 6 Appendix: NN Evaluation Using Test Material X-Ray Transmission Measurements and 50 Hidden-Layer Nodes

The results in Sec. 3 used the number of hidden-layer nodes that reduced RMSE in the cross-validation study for which results are presented in Fig. 3 (30, 30, 40, 70, and 70 nodes for the respective count-rate levels of 9%, 14%, 27%, 40%, and 50%). However, the RMSE was fairly constant when the number of hidden-layer nodes varied between 30 and 70. Here, we present the results of using 50 hidden-layer nodes for each count-rate level. Considering that the RMSE was relatively constant for a range node of selections, using less nodes may be beneficial to prevent overfitting. This study also provides some insight as to the effectiveness of a single network architecture toward the goal of developing an NN to use for a continuous range of count-rate levels.

**Fig. 9:**
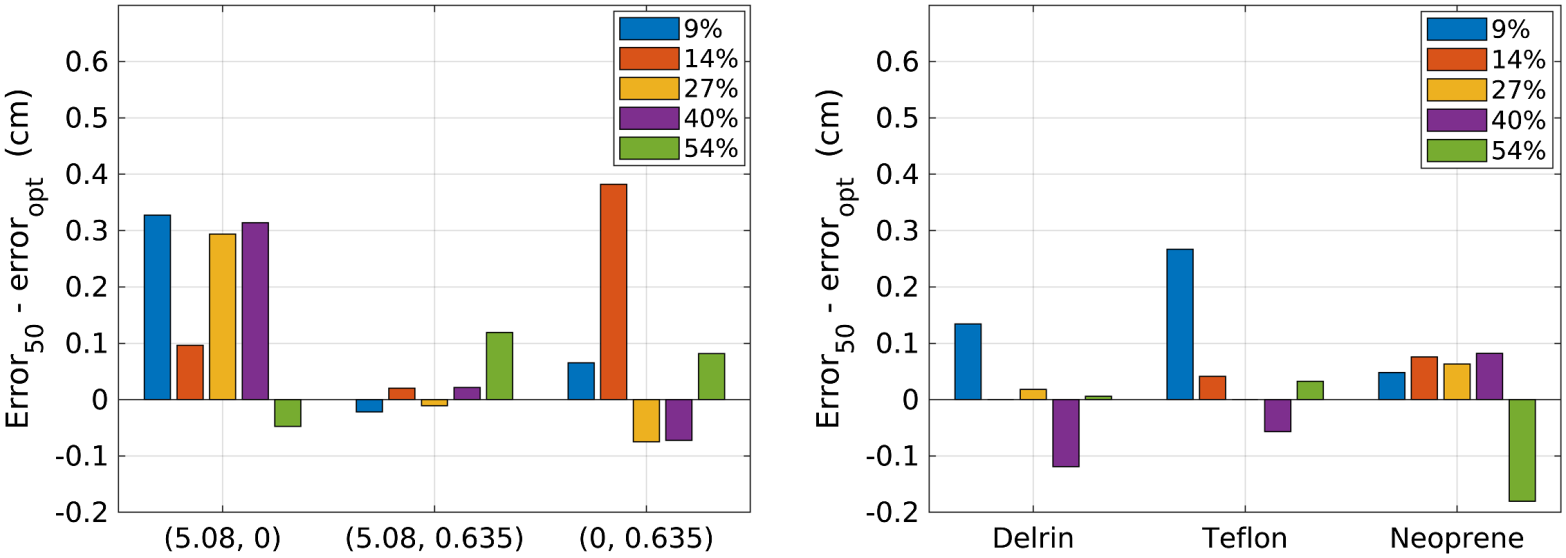
Difference in material decomposition error when using 50 hidden nodes for each count-rate level and when using the optimal number of nodes for each count-rate level: 30 (9%), 30 (14%), 40 (27%), 70 (40%), and 50 (54%). Positive values indicate improved performance by the optimal number of nodes, while negative values indicate improvement when using 50 nodes.

**Fig. 10:**
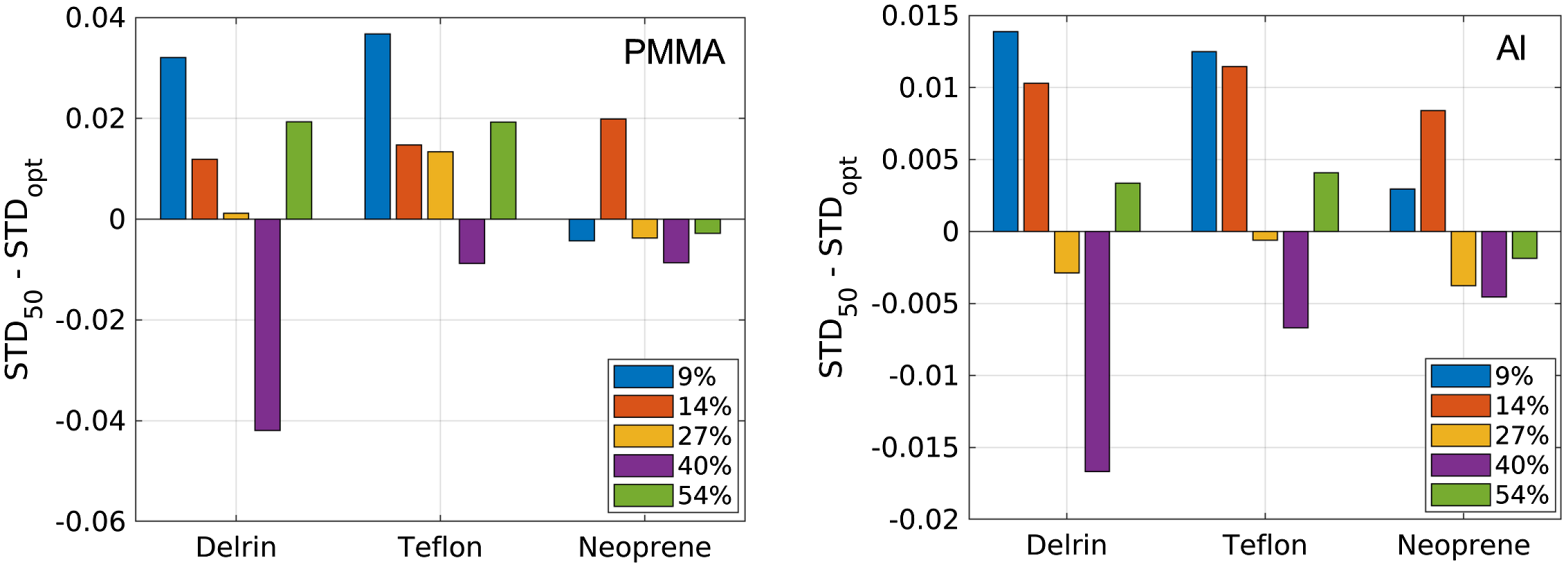
Difference in standard deviation of material decomposition estimates when using 50 hidden nodes for each count-rate level and when using the optimal number of nodes for each count-rate level: 30 (9%), 30 (14%), 40 (27%), 70 (40%), and 50 (54%). Positive values indicate improved performance by the optimal number of nodes, while negative values indicate improvement when using 50 nodes.

Figure 9 shows the difference in material decomposition errors obtained using 50 nodes and using the optimal number of nodes. Figure 10 shows the difference in the standard deviation of material decomposition estimates obtained with 50 nodes and optimal number of nodes. Using 30 nodes reduced error at the 9% and 14% count-rate levels compared with 50 nodes. For the higher count-rate levels, the benefit of using the RMSE-optimal number of nodes was not clear and varied with test material. Using 30 nodes provided lower standard deviation compared with 50 nodes at the 9% and 14% count-rate levels. Using 50 nodes reduced the standard deviation compared with 70 nodes for the 40% count-rate level. The effects of number of nodes on standard deviation varied with test material for the 27% and 14% count-rate levels. Overall, the results suggest that when the RMSE is fairly constant across a range of hidden-layer node configurations, it may be beneficial to use less nodes to reduce noise caused by overfitting. The results also support the benefit of using 30 nodes at lower count-rate levels and 50 to 70 nodes for higher count-rate levels.
